# MR-arthrography and CT-arthrography in sports-related glenolabral injuries: a matched descriptive illustration

**DOI:** 10.1007/s13244-015-0462-5

**Published:** 2016-01-08

**Authors:** Mohamed Jarraya, Frank W. Roemer, Heather I. Gale, Philippe Landreau, Pieter D’Hooghe, Ali Guermazi

**Affiliations:** Aspetar Orthopaedic and Sports Medicine Hospital, Doha, Qatar; Department of Radiology, Boston University School of Medicine, 820 Harrison Avenue, FGH Building, 3rd floor, Boston, MA USA; Department of Radiology, Mercy Catholic Medical Center, Darby, PA USA; Department of Radiology, University of Erlangen-Nuremberg, Erlangen, Germany; Department of Radiology, Massachusetts General Hospital, Harvard Medical School, Boston, MA USA

**Keywords:** Magnetic resonance imaging, Multislice computed tomography, Arthrography, Shoulder, Sports injuries

## Abstract

The combination of a large range of motion and insufficient bony stabilization makes the glenohumeral joint susceptible to injuries including dislocation in young athletes. Magnetic resonance arthrography (MR-arthrography) and computed tomography arthrography (CT-arthrography) play an important role in the preoperative workup of labroligametous injuries. This paper illustrates MR-arthrography and CT-arthrography findings acquired at the same time on the same subjects to illustrate common causes and sequelae of shoulder instability.

*Teaching Points*

• *MR-arthrography and CT-arthrography are equivalent for SLAP and full-thickness rotator cuff tears*.

• *CT-arthrography is superior in evaluating osseous defects and cartilage surface lesions*.

• *MR-arthrography is superior in evaluating intrasubstance and extra-articular tendinous injuries*.

## Introduction

The combination of a large range of motion and insufficient bony stabilization makes the glenohumeral joint susceptible to injuries, including dislocation and subsequent chronic instability especially in young active subjects [1]. Despite technological advancement of modern MRI systems, magnetic resonance arthrography (MR-arthrography) is considered the gold standard for assessment of instability and pre-operative workup for shoulder ligaments and labral injuries [2].

The shoulder joint is characterized by complex stabilizing mechanisms (static and dynamic), additional physiologic anatomic variants (e.g., sublabral foramen, Buford complex, and meniscoid labrum), and various pathologic presentations, which results in challenging imaging and even arthroscopic interpretation. Indeed, a surgical study showed relatively poor inter-rater reliability (<40 %) among orthopaedic surgeons for the exact description of anatomic structures such as the inferior glenohumeral ligament (IGHL) or the bony glenoid size during shoulder arthroscopy [3]. This fact highlights the important role of radiology in the assessment of shoulder instability using either MR-arthrography or computed tomography arthrography (CT-arthrography).

MR-arthrography and CT-arthrography yield comparable results for the detection and classification of sublabral recesses, SLAP, and full thickness rotator cuff tears [4, 5]. Although the degree of glenoid bone loss and the medial orientation of Hill Sachs lesions as detected on MR imaging has been shown to be significantly associated with engaging Hill-Sachs on physical examination [6], a study directly comparing MR-arthrography and CT-arthrography in preoperative planning of anterior shoulder instability, showed limitations of MR-arthrography in detecting glenoid rim fractures and inferior glenohumeral ligaments injuries, such as humeral avulsion of gleno-humeral ligament (HAGL). This latter entity can easily be missed during surgery depending on the surgical approach; therefore, its preoperative radiological recognition is crucial for appropriate surgical planning [7]. Also, the use of 3-D reconstruction in CT was shown to be of value for preoperative prediction of the engagement of Hill Sachs lesions, and therefore useful in surgical planning [8]. In addition to its excellent three-plane resolution, CT-arthrography is favoured by a shorter time of image acquisition yielding more comfort for the patient, and is less susceptible to motion artefacts. CT-arthrography is regarded as the reference standard for the evaluation of cartilage surface lesions [2], and is superior to MR-arthrography for preoperative planning of anterior instability [9]. On the other hand, MR-arthrography is more accurate for suspected partial thickness rotator cuff pathology [5].

While MR-arthrography is superior for the evaluation of intrasubstance ligamentous injuries and the extra-articular surface of the rotator cuff, CT-arthrography is better suited for the detection and evaluation of osseous defects (Table 1). The two modalities show comparable results otherwise, and the choice is partly based on the specific indication for each patient as well as the reader’s experience in each modality.

**Table 1:** Summary of technical recommendations and correspondent indications

Technical recommendation	Indication
Injection approach	
• Anterior	Posterior instability
• Posterior	Anterior instability
Preferred imaging modality	
• CT Arthrography	1/ Cartilage surface abnormality
	2/ Osseous defects
• MR Arthrography	1/ Intra-substance tendinous injuries
	2/ Extra-articular tendinous injuries
• MR or CT Arthrography	1/ SLAP tear
	2/ Full thickness rotator cuff tear
	3/ Labro-ligamentous complex injuries

This pictorial review aims to present MR-arthrography and CT-arthrography findings acquired at the same time on the same subjects to illustrate common causes and sequelae of shoulder instability.

## Technique

**Fig. 1:**
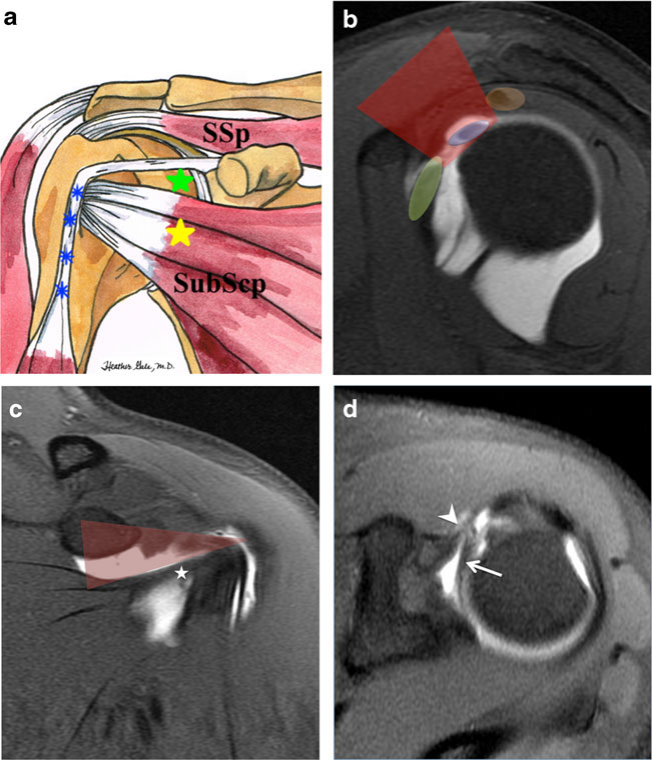
**a** Diagram showing the left shoulder in external rotation. Using the anteroinferomedial approach, the target needle crosses the subscapularis (SubScp) muscle (*yellow star*) while the rotator cuff interval (*green star*) lies between supraspinatus (SSp) and subscapularis (SubScp) muscles. The long head of the biceps tendon (*blue asterisks*) courses in the bicipital groove and is displaced laterally away from target site for the needle. **b** Sagittal T1-weighted TSE fat-suppressed MR arthrography image showing rotator interval anatomy. Anatomic structures are overlaid by different transparent colors. Rotator interval (*red*) lies between subscapularis tendon anteriorly (*green*), and supraspinatus tendon posteriorly (*orange*). Long head of biceps brachii tendon (*blue*) traverses the rotator interval on its course from the bicipital-labral anchor to the bicipital groove. **c** Coronal T1-weighted TSE fat-suppressed MR arthrography image shows rotator interval (*red transparent triangle*), inferiorly limited by subscapularis tendon (*asterisk*), and superiorly by supraspinatus tendon (not shown). **d** Axial T1-weighted TSE fat-suppressed MR arthrography through rotator interval shows long head of biceps brachii tendon (*arrow*), and coracohumeral ligament (*arrowhead*)

**Fig. 2:**
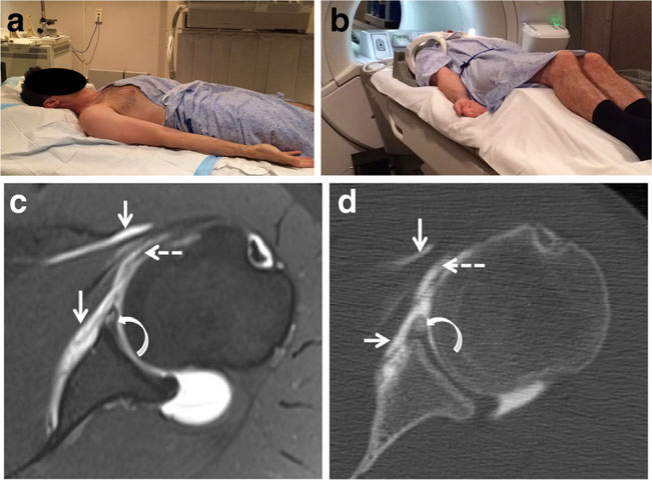
**a, b** Patient positioning. Patient is placed supine, head first, arm along the body, and the arm in external rotation for both fluoroscopy guided injection **(a)**, and subsequent MR examination **(b)**. **c**, **d** Drawbacks of the anteroinferomedial approach in shoulder CT arthrography and MR arthrography. Axial **(c)** fat-suppressed T1- and **(d)** correspondent CT - arthrography image show distortion of the subscapularis muscle with peri- (*straight arrows*) and intra-muscular (*dashed arrow*) contrast media extravasation caused by the needle traversing the subscapularis muscle. In addition, it is unclear whether intralabral contrast is also related to needle misplacement or actual tear (*curved arrow*). This can add difficulty to the interpretation. An anterior-rotator interval approach would have been optimal in this case

The shoulder joint may be approached anteriorly or posteriorly for injection. The posterior approach offers the benefit of minimizing anterior extravasation, which is particularly important when assessing suspected anterior instability. The posterior approach is reported to be more comfortable [10]; however, the anterior approach seems to be more popular with different portals described (Fig. 1). The junction of the middle and lower thirds of the humeral head is commonly targeted for arthrography injection. However this method can cause distortion of the inferior glenohumeral ligament and the anteroinferior labrum (Fig. 2). A modified anterior approach targeting the rotator cuff interval seems, therefore, to be more appropriate in suspected anterior instability [11] (Fig. 1). For intra-articular injection, both ultrasound and fluoroscopy guidance are comparable with regard to successful rate, pain score, and duration of injection, however ultrasound guidance injection has the intrinsic benefit of being non-ionizing [12].

For our study, 0.1 mL of gadoterate meglumine (Dotarem® , Guerbet, Villepinte, France) mixed with 8 mL of saline, 7 mL iodinated contrast iohexol (OmnipaqueTM 300, GE Healthcare, Princeton, NJ, USA), and 5 mL of 1 % lidocaine (final gadolinium dilution ratio of 1:200) was injected with a 22 G needle into the glenohumeral joint with fluoroscopic guidance by using an anterior approach. After the injection, gentle passive mobilization of the shoulder was performed to allow diffusion of the contrast material in the joint cavity. For both imaging modalities, patient position was the same: supine, head first, arm along the body, and the arm in external rotation.

Spiral CT was performed immediately after joint opacification by using a 40-detector helical CT scanner (Sensation 40TM, Siemens Healthcare, Erlangen, Germany). After a frontal projection scout image, a 10–15-s scanning was performed to image the volume from the top of the acromioclavicular joint to the lower margin of the axillary recess of the glenohumeral joint. Acquisition parameters were 120 kV, 315 mAs, 12-mm collimation beam, 25- cm field of view, effective pitch of 0.3, effective thickness of about 0.8 mm, and 512 × 512 matrix, bone convolution kernel (B75h); 2.0-mm slice thickness, no gap. From images acquired in the axial plane, oblique and sagittal reformations were constructed to mirror the MRI planes, using the following parameters: slice thickness = 2.0 mm, reconstruction increment = 2 mm, Kernel = B75 Sharp++, Window = bone, pitch = 0.9. CT dose length product ranged between 390 and 600 mGy cm depending on the size of the patient. X-Ray exposure (dose area product) ranged between 1.69 μGy m2 and 54.48 μGy m2.

Immediately after the CT examination, MR arthrography was performed with a 1.5 T large bore MR system (EspreeTM, Siemens Healthcare, Erlangen, Germany), using a dedicated 4-channel shoulder array coil supplied by the manufacturer. MR sequences included axial and coronal T1-weighted turbo spin echo (TR: 480 ms, TE: 13 ms, No. Excitations: 2, Field of View: 18 × 18 cm [axial] / 16 × 16 cm [coronal], imaging matrix: 256 × 192, slice thickness: 3 mm, interslice gap: 0.9 mm).

## Shoulder anatomy

**Fig. 3:**
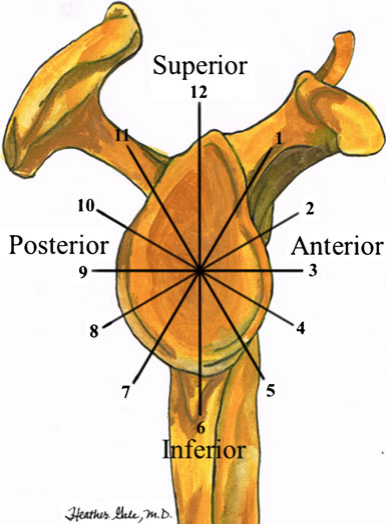
Diagram shows the clock-like division of the glenoid rim and its four sectors: inferior, posterior, anterior and superior

Compared to other joints, the bony stabilization of the glenohumeral joint is insufficient. The glenoid cavity has a teardrop (pear), or oval shape. The latter is covered by the glenoid labrum in a circular fashion forming a functional unit with the capsule, the glenohumeral ligaments, long head of the biceps, and long head of the triceps. For the purpose of localizing abnormalities, the labrum is divided in clock-wise fashion (Fig. 3). The labrum is likened to the face of a clock, with the superior portion positioned at 12 o’clock and the inferior portion at 6 o’clock. By convention, the anterior portion is positioned at 3 o’clock and the posterior portion at 9 o’clock for both shoulders [13].

**Fig. 4:**
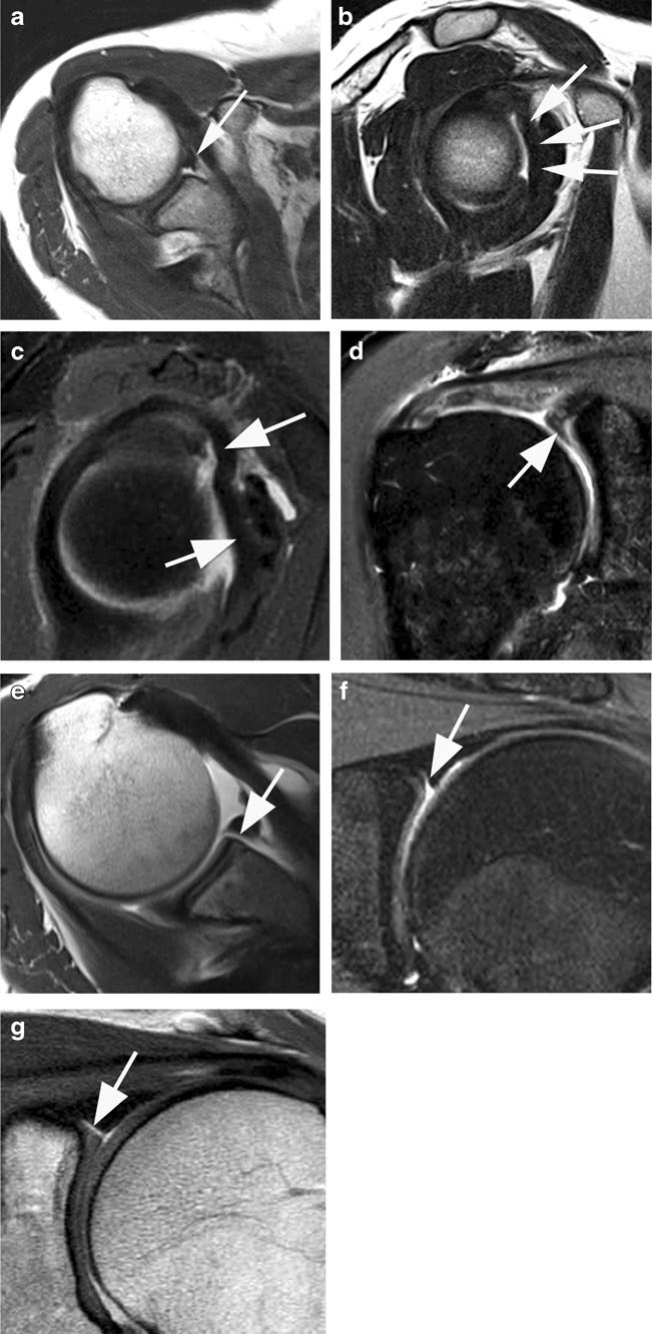
Normal variants around the superior labrum and the biceps anchor. **a, b** Axial **(a)** and sagittal **(b)** non fat-suppressed proton density-weighted images show a missing anterior superior labrum. The middle gleno-humeral ligament appears thick and of cord-like appearance representative of a so-called Buford-complex (*arrows*). **c** Another example of a Buford complex on a fat-suppressed proton density-weighted image *(arrows)*. **d** Coronal fat-suppressed proton-density-weighted image shows a meniscoid shaped labrum (*arrow*). **e** Axial T1-weighted MR arthrography image shows a so-called sublabral foramen (*arrow*). **f, g** The attachment of the biceps tendon at the glenoid may show different appearances. Proton density-weighted fat suppressed **(f)** and nonfat-suppressed **(g)** images of two different patients show a so-called Type II bicipito-labral complex representing a recess at the labral attachment (*arrows*). Such recesses point medially and as such may be distinguished from so-called SLAP II lesions that are commonly pointing towards the lateral aspect of the biceps anchor/proximal tendon

At its anterosuperior portion, the glenoid labrum anchors the to biceps tendon, called the labor-bicipital complex. Normal variants include the sublabral recess and foramen [13]. The Buford complex is rare and consists in the absent anterosuperior labrum replaced by a cordlike middle glenohumeral ligament (MGHL). The inferior part of the labrum anchors to the IGHL. Normal variants are optimally seen on axial and coronal images of CT and MR-arthrography [14] (Fig. 4).

The capsule consists of a complex system of circularly and radially arranged collagen fibres and is strengthened by multiple reinforcements, including the superior glenohumeral complex (SGHL), the MGHL, and the IGHL. The rotator interval is defined as the space between the supraspinatus and subscapularis muscles.

**Fig. 5:**
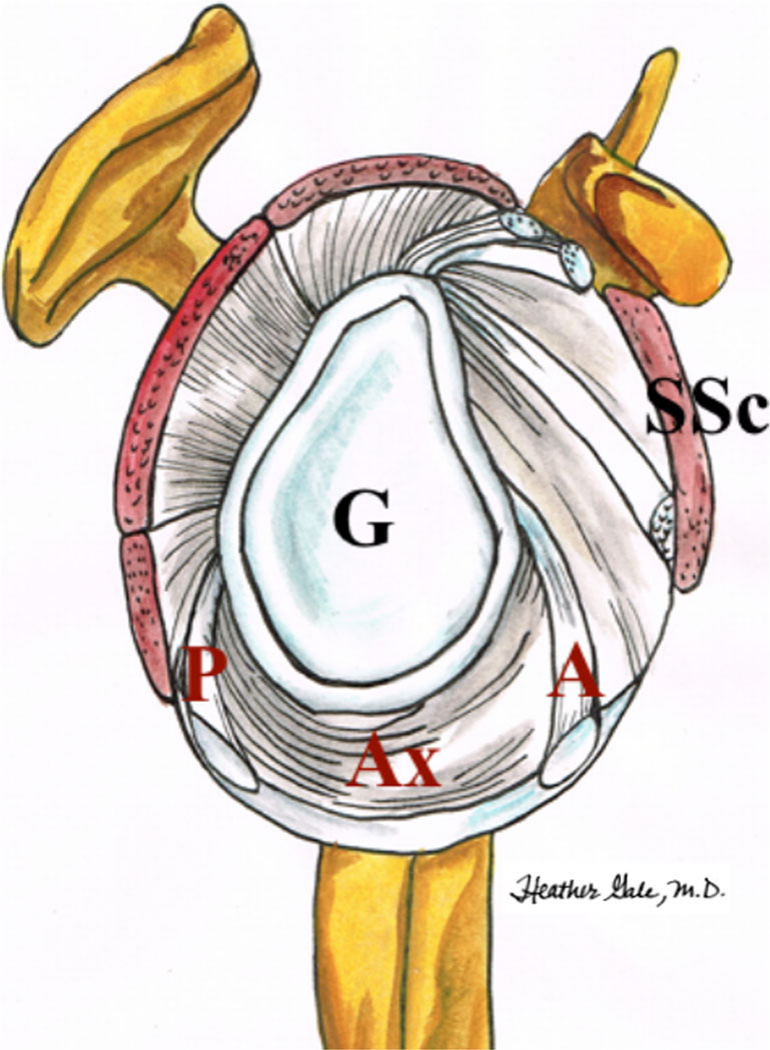
Diagram shows the inferior glenohumeral ligament with its anterior (*A*), posterior bands (*P*) and axillary pouch (*Ax*). *G* glenoid cavity, *SSc* Subscapularis muscle

**Fig. 6:**
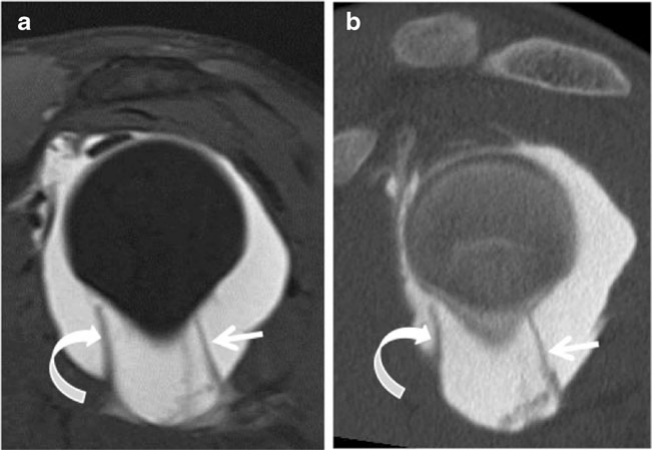
Inferior glenohumeral ligament. Sagittal (**a**) fat-suppressed T1-weighted MR-arthrography and (**b**) corresponding CT - arthrography images show the anterior band (*curved arrow*) and posterior band (*straight arrow*) of the inferior glenohumeral ligament

The MGHL is variably developed and radiates from the superior glenoid tubercle to the inferior parts of the lesser tuberosity [15]. The IGHL consists of a strong anterior band and posterior band with the axillary recess residing between them, and is also called a hammock (Figs. 5, and 6). Both parts of the ligament stabilize the humeral head in the vulnerable abduction and external rotation movements [15].

## Superior labral anteroposterior (SLAP) tears

**Fig. 7:**
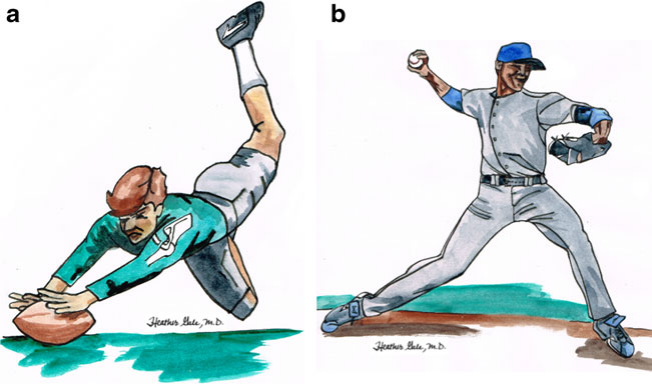
Mechanisms of injury of SLAP lesions: **a** Acute from fall on outstretched arm (soccer goalkeeper, rugby player, etc.), or **b** chronic from superior traction to the biceps tendon from overhead sports (baseball pitcher)

SLAP lesions are defined as a pathologic labral separation from the biceps anchor, with variable extension to either the anterior or posterior portion of the labrum. Determining the type of attachment of the biceps to the superior labrum and adjacent supraglenoid notch, as well as the presence of anatomic variations, is the first step in accurate evaluation of this region. SLAP tears are commonly found in throwing athletes because of the high stresses of repetitive overhead throwing, which results in excessive traction to the biceps tendon. Snyder found that the mechanism of injury most commonly was related to an acute trauma: fall on an outstretched arm with the shoulder positioned in abduction and slight forward flexion at the time of impact [16]. Andrews et al. reported high prevalence of SLAP lesions among overhead throwing athletes (baseball pitchers) suggesting a chronic injury [17] (Fig. 7).

**Fig. 8:**
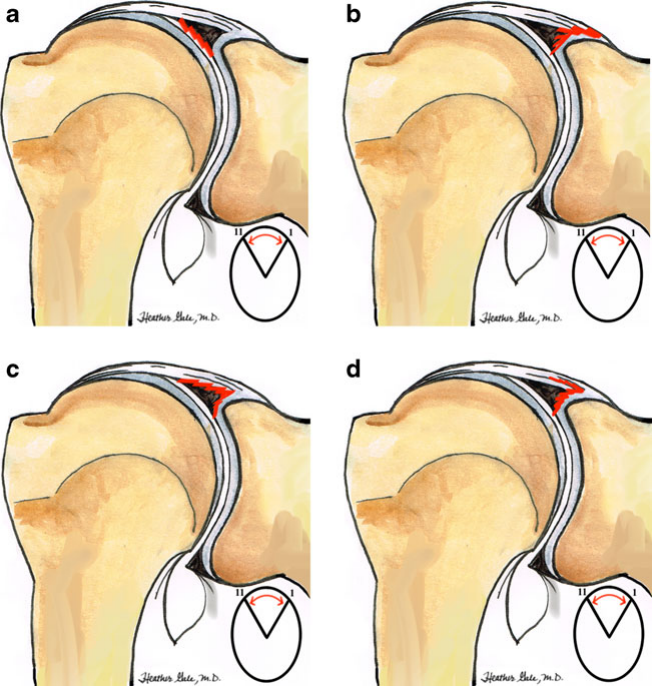
Drawing shows four main types of SLAP injury. **a** Type I. Fraying of the free edge of the superior labrum, intact biceps tendon (degenerative) (11–1 o’clock). **b** Type II. Most common type. Avulsion of superior labrum and biceps anchor (11-1o’clock). **c** Type III. Bucket-handle tear of the superior labrum with intact biceps anchor (11–1 o’clock). **d** Type IV. Extension of a bucket-handle of the superior labrum into biceps anchor (11–1 o’clock)

**Fig. 9:**
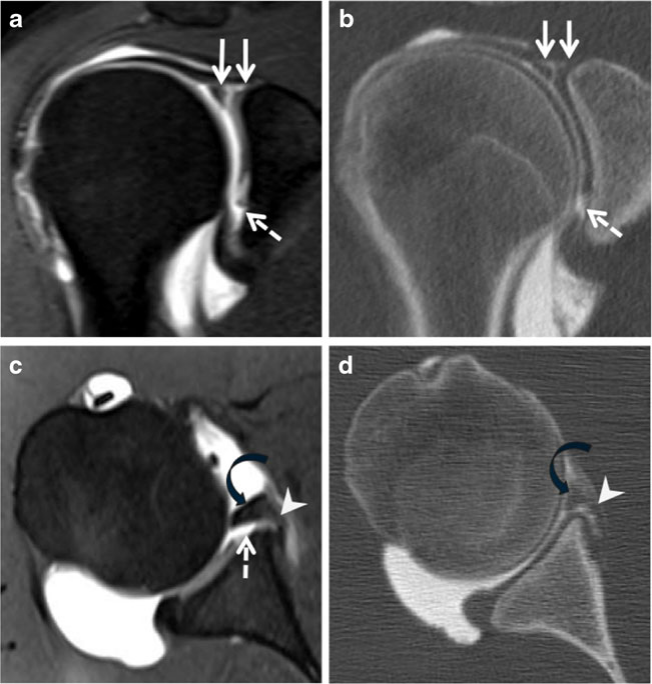
Bucket-handle SLAP tear (Type 3) with Perthes lesion in a 23-year-old male athlete with a one-month history of right shoulder pain after a fall onto outstretched arm. **a** Coronal and **c** axial fat-suppressed T1-weighted MR arthrography with correspondent **b** coronal and **d** axial CT arthrography images. Presence of a Bucket handle tear of the labro-bicipital complex extending across the bicipital attachment (*white plain arrows*) and to the anteroinferior labrum (*11*–*6 o’clock*). The anteroinferior labrum is avulsed (*black curved arrow*) with medial stripping of an intact scapular periosteum (*white arrowhead*) consistent with a Perthes lesion. There is a cartilage defect of the anteroinferior labrum (*dashed arrow*). These findings can be classified either as a SLAP III (with regard to the bucket handle tear) with Perthes lesion, or SLAP V (with regard to the anteroinferior extension of the labral tear)

**Fig. 10:**
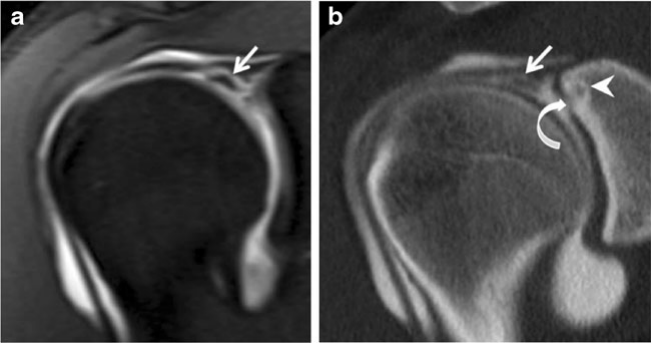
SLAP tear type IV in a 20-year-old goalkeeper after a fall onto fully abducted right shoulder with immediate pain. Coronal (**a**) fat-suppressed T1-weighted MR arthrography and (**b**) corresponding coronal CT arthrography image. Bucket handle tear of the labro-bicipital complex extending across the bicipital attachment (*straight arrow*). Note the presence of a small focal cartilage defect of the superior labrum only detected on the CT arthrography image (*curved arrow*) with adjacent subchondral cyst (*arrowhead*) suggesting a chronic chondropathy

**Fig. 11:**
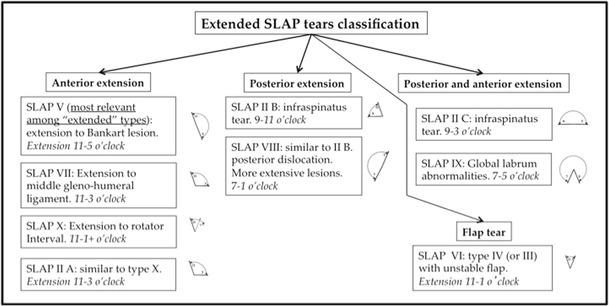
Diagram showing extended SLAP tear classification according to direction of extension of labral tear [13, 19, 20]

**Fig. 12:**
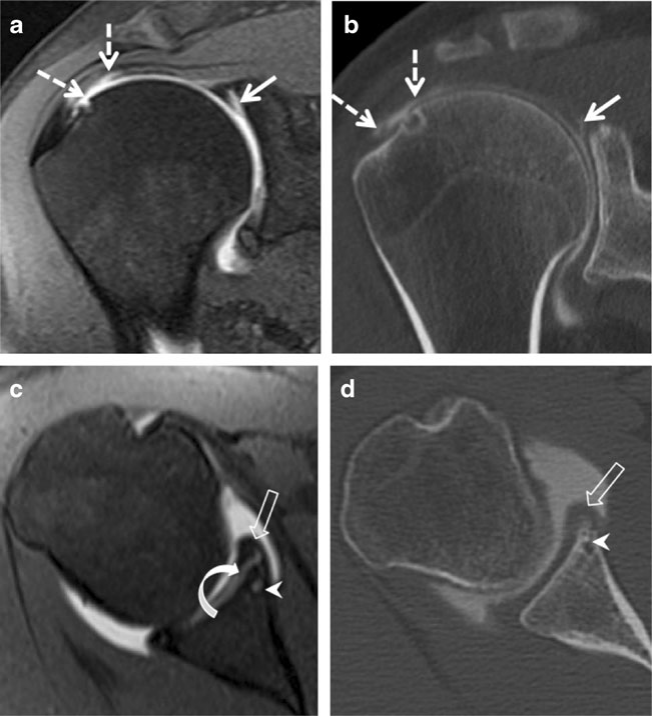
SLAP tear type V with rotator cuff lesion in a 38-year-old male athlete with right shoulder pain. **a** Coronal and **c** axial fat-suppressed T1-weighted MR arthrography and correspondent **b** coronal and **d** axial CT arthrography images show an intermediate undersurface tear of the anterior portion of the supraspinatus (*dashed arrows*). Note also the presence of a labro-bicipital tear extending across the biceps attachment (*curved arrow*) and inferiorly (*11*–*6 o’clock*). Hypertrophy (*empty arrow*) and linear tear (*curved arrow*) of the inferior labrum communicating with a small paralabral cyst (*arrowhead*) are noted. This constellation of findings is in keeping with SLAP V lesion

**Fig. 13:**
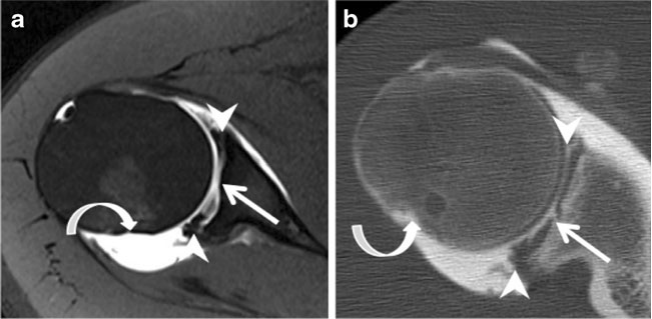
Global labral (SLAP IX) tear in a 24-year-old male athlete with a 10-month history of right shoulder pain. Axial (**a**) fat suppressed T1-weighted MR arthrography and (**b**) corresponding axial CT arthrography images. Cartilage defect (*straight arrow*) is more conspicuous on CT arthrography than on the MR arthrography image. Circumferential labral abnormalities (*arrowheads*) extending across the labro-bicipital complex (not shown) in keeping with SLAP tear type IX. A subtle osseous impaction of the posterosuperior aspect of the humeral head (Hill-Sachs defect) (*curved arrow*) is detected suggesting prior anterior dislocations

SLAP tears were initially classified into four types (Fig. 8) [16]: Type I: fraying of the free edge of the superior labrum with a normal biceps anchor is thought to be a degenerative process and uncommon source of pain [18]. Type II: fraying of the free edge of the superior labrum associated with a detached biceps anchor from the superior glenoid tubercule, is the most commonly encountered during arthroscopy. Type III: bucket handle tear of a meniscoid superior labrum with an otherwise normal biceps tendon attachment (Fig. 9). Type IV: bucket handle tear of a meniscoid superior labrum with extension of the tear into the biceps tendon (Fig. 10). Other later types have been introduced and are summarized in Fig. 11 [13, 19, 20]. For instance, SLAP Type V (Fig. 12) is defined by associating a SLAP tear with a Bankart type labral lesion, while type IX is defined by a global labral injury (Fig. 13).

## Anterior-inferior labro-ligamnetous injuries

The most common labral lesion is the classic labral Bankart lesion, in which the anterior-inferior labrum is separated from the glenoid. The anterior labroligamentous periosteal sleeve avulsion (ALPSA) occurs after multiple dislocations and consists in anteroinferior labral avulsion leading to medial displacement and inferior rotation of the labroligamentous complex with intact periosteum, in a sleeve like fashion [21].

The Perthes lesion is a variation of the Bankart lesions and occurs when the scapular periosteum remains intact but is stripped medially and the anterior labrum is avulsed from the glenoid but remains partially attached to the scapula by the intact periosteum [21] (Fig. 9).

The glenolabral articular disruption (GLAD) is a superficial tear of the anteroinferior labrum associated with an articular cartilage lesion of the anterior inferior quadrant of the glenoid. The injury is thought to result from glenohumeral impaction while the arm is abducted and externally rotated [21].

The HAGL lesion consists in an isolated tear of the IGHL at its humeral insertion following vigorous shoulder dislocation. HAGL may be caused by traumatic hyperabduction with external rotation of the arm. Different activities may be involved, such as rugby, snow or water skiing, surfing, football, volleyball, basketball, ice hockey, wrestling, and boxing [22]. This type of injury represents a pitfall at both arthroscopy and open shoulder surgery, since it can easily be overlooked if the humeral neck is not specifically searched for this finding. It is therefore paramount that radiologists be aware of this entity for an accurate preoperative diagnosis. Contrast extravasation can be observed at the insertion site [21].

The Hill-Sachs lesion is a posttraumatic impaction injury along the posterolateral aspect of the humeral head, resulting from repetitive anterior glenohumeral subluxation (Fig. 13).

## posterior glenuhumeral injuries

**Fig. 14:**
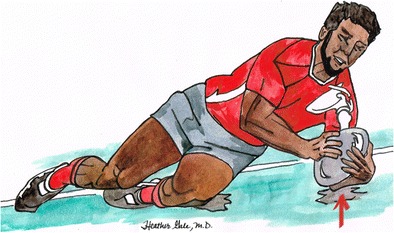
Drawing shows the mechanism of posterior shoulder injury in rugby player falling on a flexed elbow and shoulder (forced adduction and internal rotation)

**Fig. 15:**
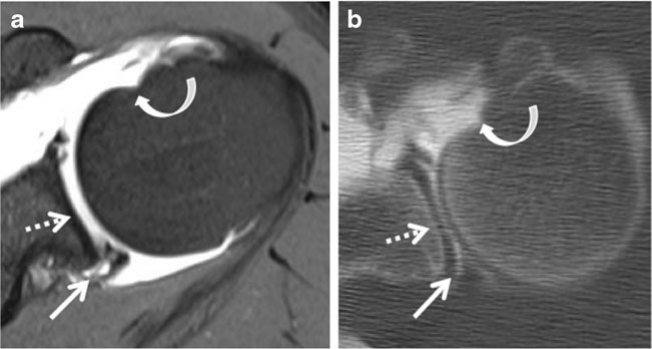
Posterior instability in a 32-year-old male athlete following a ski fall on a flexed elbow and shoulder a year earlier. Axial (**a**) fat-suppressed T1-weighted MR arthrography and (**b**) corresponding CT arthrography images. Posterior labral tear consistent with reverse Bankart tear (*straight arrow*). Cartilage thinning of the glenoid (*dashed arrow*) and anterosuperior humeral head impaction (*curved arrow*), consistent with reversed Hill-Sachs. The labral injury extends to the superior labrum (not shown)

Posterior labral tears typically result from a single event of axial loading on the arm while adducted, flexed, and internally rotated (Fig. 14), or from repetitive trauma, such as straight arm pass blocking in football, or bench pressing [23–25]. The patterns of injury are usually the reverse of those found following anterior dislocation. Tears that occur in the posterior labrum are referred to as reverse Bankart, whereas impaction of the anterosuperior humeral head gives rise to the reverse Hill Sachs defect (Fig. 15).

Kim classified labral injuries associated with posterior instability into three categories: separation without displacement, incomplete avulsion (concealed avulsion arthroscopy), and loss of contour, either from a flap tear or a chondrolabral erosion [26].

## Conclusion

Shoulder CT-arthrography and MR-arthrography yield very similar results in many clinical scenarios. Definition of the respective indications of both techniques depends on many factors, but it is primarily the relative availability and local experience. MR-arthrography is superior for visualization of the intra-articular portion of ligaments and depiction of bone marrow changes, while CT-arthrography is the method of choice for assessment of articular cartilage surface lesions, fractures, and bony avulsions like osseous Bankart lesions.
